# The clinical importance of tumour-infiltrating macrophages and dendritic cells in periampullary adenocarcinoma differs by morphological subtype

**DOI:** 10.1186/s12967-017-1256-y

**Published:** 2017-07-03

**Authors:** Sebastian Lundgren, Emelie Karnevi, Jacob Elebro, Björn Nodin, Mikael C. I. Karlsson, Jakob Eberhard, Karin Leandersson, Karin Jirström

**Affiliations:** 1 Department of Clinical Sciences Lund, Oncology and Pathology, Lund University, 221 85 Lund, Sweden; 2 Department of Microbiology, Tumor and Cellbiology, Karolinska Institute, 171 76 Stockholm, Sweden; 3 Department of Translational Medicine, Center for Molecular Pathology, Lund University, Lund, Sweden

## Abstract

**Background:**

Dendritic cells (DC) and tumour-associated macrophages (TAM) are essential in linking the innate and adaptive immune response against tumour cells and tumour progression. These cells are also potential target for immunotherapy as well as providing a handle to investigate immune status in the tumour microenvironment. The aim of the present study was to examine their impact on prognosis and chemotherapy response in periampullary adenocarcinoma, including pancreatic cancer, with particular reference to morphological subtype.

**Methods:**

The density of tolerogenic immature CD1a^+^ dendritic cells (DC), and MARCO^+^, CD68^+^ and CD163^+^ tissue-associated macrophages (TAM) was analysed by immunohistochemistry in tissue micro arrays with tumours from 175 consecutive cases of periampullary adenocarcinoma who had undergone pancreaticoduodenectomy, 110 with pancreatobiliary type (PB-type) and 65 with intestinal type (I-type) morphology. Kaplan–Meier and Cox regression analyses were applied to determine the impact of immune cell infiltration on 5-year overall survival (OS).

**Results:**

High density of CD1a^+^ DCs was an independent prognostic factor for a reduced OS in PB-type but not in I-type tumours (adjusted HR = 2.35; 95% CI 1.13–4.87). High density of CD68^+^ and CD163^+^ TAM was significantly associated with poor OS in the whole cohort, however only in unadjusted analysis (HR = 1.67; 95% CI 1.06–2.63, and HR = 1.84; 95% CI 1.09–3.09, respectively) and not in strata according to morphological subtype. High density of MARCO^+^ macrophages was significantly associated with poor prognosis in I-type but not in PB-type tumours (HR = 2.14 95% CI 1.03–4.44), and this association was only evident in patients treated with adjuvant chemotherapy. The prognostic value of the other investigated immune cells did not differ significantly in strata according to adjuvant chemotherapy.

**Conclusions:**

The results from this study demonstrate that high infiltration of tolerogenic immature DCs independently predicts a shorter survival in patients with PB-type periampullary adenocarcinoma, and that high density of the MARCO^+^ subtype of TAMs predicts a shorter survival in patients with I-type tumours. These results emphasise the importance of taking morphological subtype into account in biomarker studies related to periampullary cancer, and indicate that therapies targeting dendritic cells may be of value in the treatment of PB-type tumours, which are associated with the worst prognosis.

**Electronic supplementary material:**

The online version of this article (doi:10.1186/s12967-017-1256-y) contains supplementary material, which is available to authorized users.

## Background

Periampullary adenocarcinoma, including pancreatic cancer, is a heterogenous group of tumours that originate in the area around the caput pancreatitis and the periampullary region. They have a dismal prognosis due to lack of symptomatology until late stages and lack of effective conventional therapy. Today, the 5-year survival rate for patients with pancreatic cancer is 7% and the positive survival trends seen in other major cancer types have not yet been observed in these patients [1]. Recent research has started to explore the significance of the immune system and components of the inflammatory tumour microenvironment as potential novel treatment targets in a variety of solid cancers, including pancreatic cancer.

Professional antigen presenting cells (APC) such as dendritic cells (DC) and macrophages play a pivotal role in tumorigenesis and in the complex microenvironment of pancreatic cancer and periampullary adenocarcinomas [2]. Tumour associated macrophages (TAM) can roughly be divided into two subtypes, M1 and M2, whereof M1 are associated with pro-inflammatory properties and M2 anti-inflammatory properties. TAMs show a complex phenotypic and behavioural variation and have the ability to promote angiogenesis, invasion, metastasis and regulate of inflammation [3–5]. Although the M1–M2 polarization model may be useful, it should be pointed out that TAMs exist on a spectrum, exhibiting potent spatiotemporal plasticity in regard to phenotype [6]. Previous research in pancreatic cancer has shown that the presence of M2 polarized TAMs is associated with poor prognosis [7]. Another study could only establish a prognostic value to M2 TAMs, and not to the pan-macrophage tissue resident population [8]. Further, TAMs, both CD68^+^ and CD163^+^, located in the stromal compartment have been shown to have prognostic value in breast cancer, illustrating that the localisation of TAMs within the histological architecture is relevant [9].

The macrophage receptor with collagenous structure (MARCO) is a scavenger receptor involved in the recognition of pathogens through pathogen associated molecular patterns (PAMPs) [10, 11]. MARCO is expressed by both activated DCs and a restricted population of tissue resident macrophages, and besides playing an essential role in recognising PAMPs, it is also involved in migration capacity [10]. In addition, there is also evidence of a modulating role of MARCO on Toll-like receptors (TLRs) and thus the innate immune response to pathogens [12, 13]. Due to its functions and interactions, MARCO has been put forward as a potential novel immunotherapy target and it was recently shown to reduce tumour growth in mouse models of cancer as well as adding to the effect of checkpoint therapy [10, 14]. As of yet, the prognostic and potential predictive role of MARCO^+^ TAMs have neither been described in periampullary adenocarcinoma nor in pancreatic cancer.

As with TAMs, tumour infiltrating DC (TIDC) show a complex phenotypic variation and has the capacity to interact with many of the plethora of cells present in the tumour microenvironment [15]. High TIDC density has been shown to correlate with an improved prognosis in pancreatic cancer [16], however another study could not establish any association between survival and TIDC density due to scarcity of infiltration [17]. TIDCs have previously been reported to be either correlated with poor prognosis in cancer when expressing the immature DC marker CD1a, or to be beneficial for prognosis when expressing the mature DC markers DC-LAMP or CD83 [18]. It should be noted that cutaneous dendritic cells, such as Langerhans cells, express CD1a even throughout maturation [18].

There is accumulating evidence that the morphological subtype of periampullary adenocarcinoma is of larger importance than the anatomical origin, with pancreatobiliary-type (PB-type) tumours having a worse prognosis than intestinal-type (I-type) tumours [19]. Thus, studies related to the prognostic and predictive role of components of the tumour microenvironment in periampullary adenocarcinoma need to take morphology into consideration. As of yet, we are not aware of any studies that have investigated the density and prognostic significance of TAMs, TIDCs or subtypes of these in relation to morphological subtype in periampullary adenocarcinoma. Therefore, the aim of this study was to explore the clinicopathological correlates and prognostic impact of tumour-infiltrating macrophages (CD68^+^, CD163^+^ and MARCO^+^) and tolerogenic immature DCs (CD1a^+^) in a clinically well-annotated consecutive cohort of periampullary adenocarcinoma, with particular reference to morphological subtype and adjuvant chemotherapy.

## Methods

### Study cohort

The study cohort is a retrospective consecutive series consisting of all primary tumours from 175 patients with periampullary adenocarcinoma that underwent pancreaticoduodenectomy at the University hospitals of Lund and Malmö, Sweden, from January 1, 2001 to December 31, 2011. Follow-up began at the date of surgical treatment and had the following end points: date of death, 5 years after surgery or on the 31 of December 2013. Vital status information was obtained from the Swedish National Civil Register. Data on adjuvant treatment was obtained from patient charts where 77 patients (44%) received adjuvant chemotherapy (gemcitabine = 52; gemcitabine–capcitabine = 4; 5-FU = 13; 5-FU-oxaliplatin = 5 and gemcitabine–oxaliplatin = 3), and 98 patients (56%) did not receive any adjuvant chemotherapy. All cases underwent histopathological re-evaluation, whereby 110 cases were classified as being of PB-type and 65 cases as being of I-type [20]. Data on CD56^+^ and CD3^+^ lymphocyte infiltration has been previously described [21].

### Tissue microarray construction and immunohistochemistry

Tissue microarrays (TMA) were constructed as previously described [22, 23], using a semi-automated arraying device (TMArrayer, Pathology Devices, Westminister, MD, USA). A set of three 1 mm cores was obtained from viable, non-necrotic areas of the primary tumours. For immunohistochemical (IHC) analysis of CD1a, CD68, CD163 and MARCO, 4 μm TMA-sections were pre- treated using ULTRA Cell Conditioning Solution 1, pH 8.5 (Ventana Medical Systems Inc., Tucson, AZ, USA) for heat induced epitope retrieval, and stained in a Ventana BenchMark stainer (Ventana Medical Systems Inc.) with the following antibodies: CD1a: clone NCL-CD1a-220, diluted 1:25, LEICA Biosystems, Newcastle, UK, CD68: clone KP1, diluted 1:1000, Dako, Glostrup, Denmark, CD163: clone 10D6 diluted 1:200 Novus Biologicals, Abingdon, United Kingdom, MARCO clone HPA063793, diluted 1:250, Atlas Antibodies, Bromma, Sweden. The antibody-antigen complex was visualized with ultraView Universal DAB Detection kit (Ventana Medical Systems Inc.).

### Assessment of immunohistochemistry

Immune cells positive for the TAM markers CD68, CD163 and MARCO, and for the tolerogenic immature DC marker CD1a were counted manually in each TMA core. Immune cell location for CD68, CD163, and CD1a staining within the tissue landscape was denoted and classified as either stromal or in the tumour nest (defined as being juxtaposed to a tumour cell or in the direct vicinity of a tumour cell).

### Statistics

A median of all cores was calculated and used in subsequent statistical analyses. Mann–Whitney U test was used to assess differences in distribution of immune cell infiltration in relation to established clinicopathological factors. Classification and regression tree analysis (CRT) was applied in order to find an optimal prognostic cut-off between high and low immune cell density. Two patients with PB-type adenocarcinoma received neoadjuvant chemotherapy and were excluded from the statistical analyses. In addition, three patients were excluded from the survival analyses, two with I-type adenocarcinomas on the basis of death due to complications after surgical treatment, and one with PB-type adenocarcinoma on the basis of emigration. Paired T test was used to illustrate associations of immune cell infiltration signatures. Kaplan–Meier analysis and log rank test were applied to illustrate difference in 5-year overall survival (OS). Numbers at risk was used to illustrate the number of patients at risk of death at given intervals during the 5 year follow-up. Cox regression proportional hazard models were used to estimate hazard ratios (HR) in both univariable and multivariable analysis, adjusted for T-stage, N-stage, differentiation grade, infiltration in vascular, lymphatic and perineural tissue, age and adjuvant chemotherapy.

All calculations were performed using IBM SPSS Statistics for Mac version 24.0 (IBM, Armonk, NY, USA). All statistical tests were two-sided and p values <0.05 were considered significant.

## Results

### Distribution and intercorrelation of the investigated immune cells

**Fig. 1:**
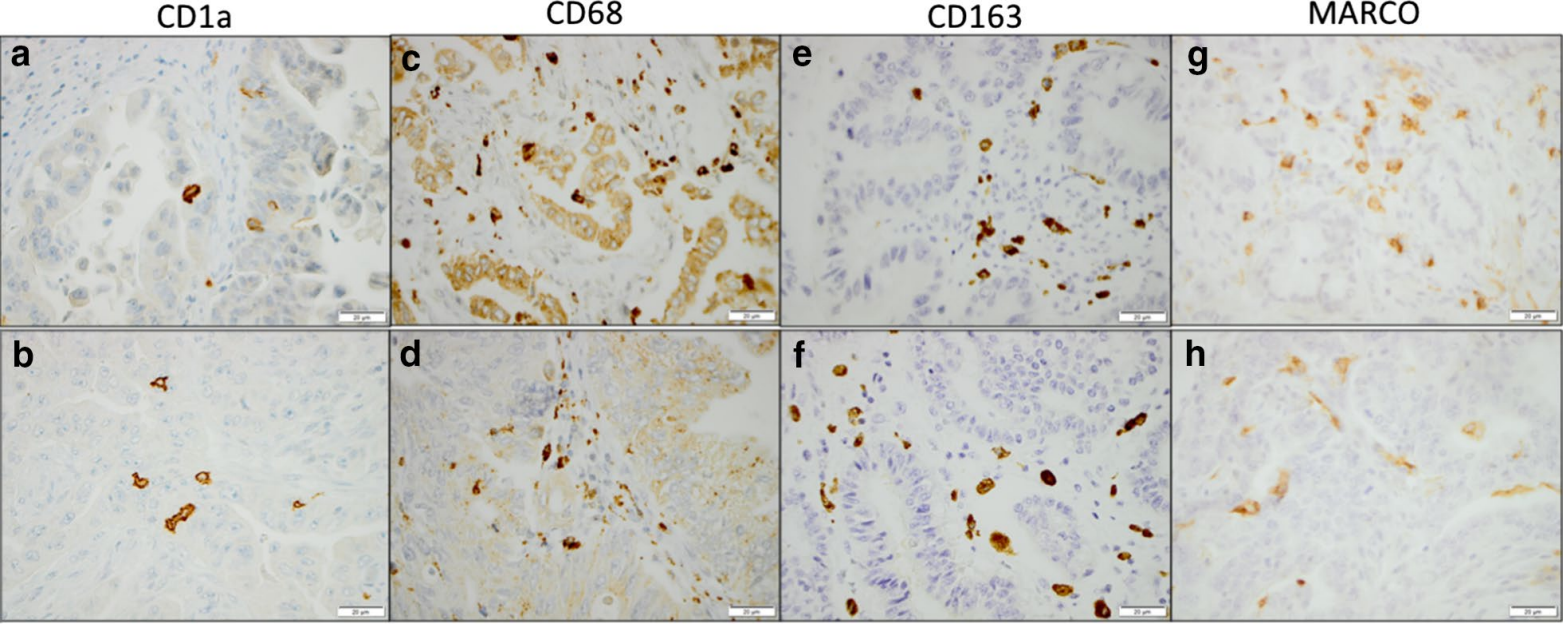
Sample immunohistochemical images. Sample images of immunohistochemical staining of CD1a^+^ TIDC in **a** PB-type, **b** I-type tumour, CD68^+^ TAM in **c** PB-type, **d** I-type tumour, CD163^+^ TAM in **e** PB-type, **f** I-type tumour, and MARCO^+^ TAM in **g** PB-type, **h** I-type. *Scale bar* represents 20 μm

CD1a^+^ tolerogenic immature dendritic cells could be assessed in 173 (98.9%) cases, CD68^+^ macrophages in 166 (94.9%) cases., CD163^+^ macrophages in 167 (95.4%) cases, and MARCO^+^ macrophages could be assessed in 172 (98.3%) cases. Sample immunohistochemical images are shown in Fig. 1. As shown in Table 1, paired T test revealed that there was a significant correlation between infiltration of CD68^+^ and CD163^+^ cells (p = 0.001) and CD68^+^ and MARCO^+^ cells (p = 0.001, Table 1). Adding to this, there were significant associations between CD3^+^ lymphocyte infiltration and CD68^+^ and CD163^+^ TAM infiltration, respectively (p = 0.005 and p < 0.001, Table 1). Further, there were significant associations between CD1a^+^ TIDC and CD68^+^ TAMs with CD56^+^ NK/NKT cell infiltration, respectively (p = 0.007 and p = 0.033, Table 1). There was however no significant correlation between CD1a^+^ TIDC and TAM infiltration, nor with CD3^+^ lymphocyte density (Table 1).

**Table 1:** Relationship between immune cell-specific markers

	CD1a	CD68	CD163	CD3	CD56
CD1a					
R		0.094	0.082	0.081	0.217^a^
p		0.231	0.294	0.294	0.007
n		164	165	171	155
CD68					
R	0.094		0.266^b^	0.217^a^	0.174^a^
p	0.231		0.001	0.050	0.033
n	164		161	164	150
CD163					
R	0.082	0.266^b^		0.366^b^	0.080
p	0.294	0.001		<0.001	0.329
n	165	161		165	151
MARCO					
R	0.081	0.274^b^	0.074	0.092	−0.038
p	0.298	0.001	0.346	0.234	0.298
n	166	164	164	170	170

*R* Spearman’s correlation coefficient, *p* p value, *n* number of cases available for analysis

^a^Significance at the 5% level

^b^Significance at the 1% level

### Associations of immune cell density with clinicopathological characteristics

The associations of immune cell density with patient and tumour characteristics are shown in Additional files 1, 2, 3 and 4. There were no significant associations between CD1a^+^ DCs infiltration and clinicopathological factors (Additional file 1). High infiltration of CD68^+^ TAM was significantly higher in males (p = 0.044) in I-type tumours and with absent vascular tumour growth (p = 0.018) in PB-type tumours (Additional file 2). There were no significant associations between CD163^+^ TAM infiltration and any clinicopathological characteristics (Additional file 3). Interestingly, high MARCO^+^ expression was significantly higher in tumours with absent vascular growth (p < 0.001) in PB-type tumours (Additional file 4).

### Prognostic significance of CD1a^+^ TIDCs

**Fig. 2:**
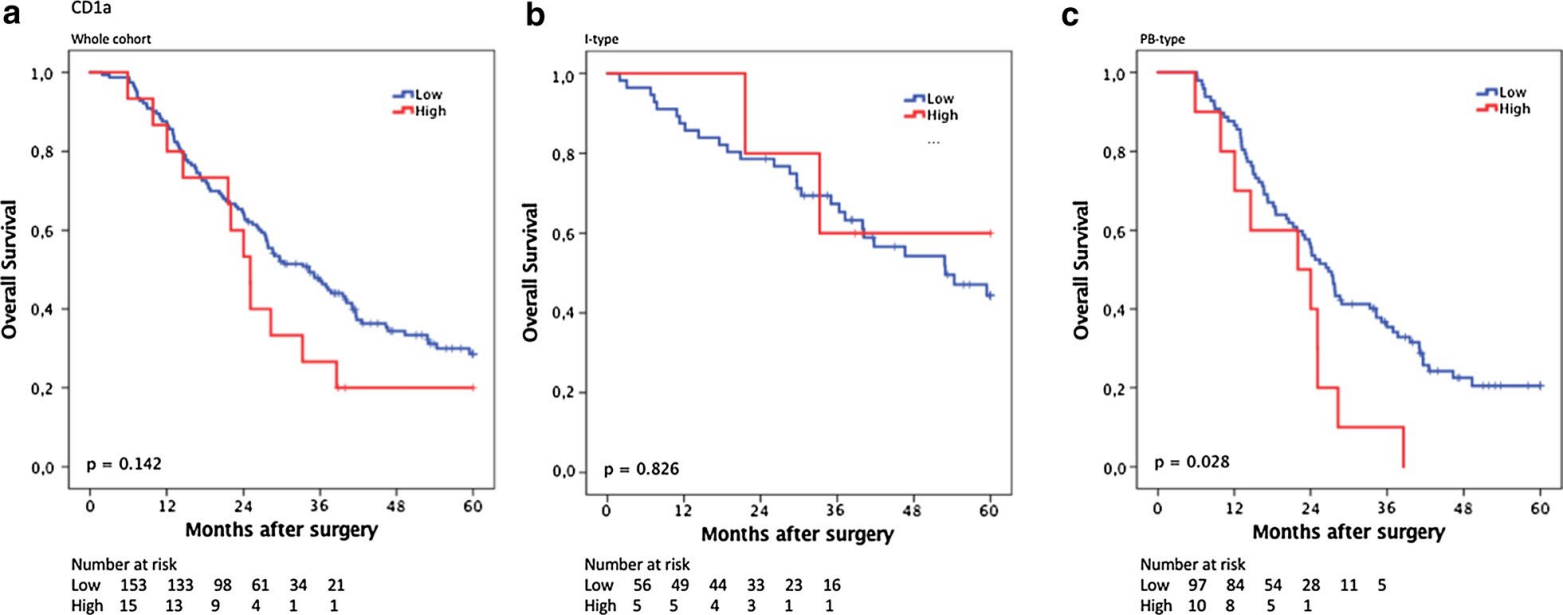
Kaplan–Meier estimates of survival according to CD1a^+^ TIDC density. Kaplan–Meier estimates of 5-year overall survival according to high and low TIDCs density in **a** the entire cohort, **b** in I-type tumours and **c** in PB-type tumours

CRT analysis established an optimal prognostic cut off for total CD1a^+^ cell infiltration at 2.75, whereby 158 cases were classified as having high DC density and 15 cases as having low DC density. As shown in Fig. 2, Kaplan–Meier analysis in the whole cohort and in I-type tumours did not demonstrate any significant association between OS and DC infiltration. However, in PB-type tumours, a significantly shorter OS was demonstrated for patients with high DC infiltration (p = 0.028, Fig. 2). As shown in Table 2, this association was confirmed in unadjusted Cox regression analysis (HR = 2.09; 95% CI 1.07–4.09; p = 0.031) and remained significant in adjusted analysis (HR = 2.35; 95% CI 1.13–4.87; p = 0.022). Similar, but non-significant, trends towards an association with survival were seen in PB-type tumours when CD1a^+^ DC density in stroma or tumour-nest was analysed separately (data not shown). The prognostic significance of CD1a^+^ DCs did not differ significantly according to adjuvant chemotherapy (data not shown).

**Table 2:** Cox proportional hazards analysis of the impact of investigated cell populations on overall survival according to morphology

	Whole cohort			I-type			PB-type		
	N events	HR (95% CI)	P	N events	HR (95% CI)	P	N events	HR (95% CI)	P
CD1a^+^ TIDC									
Univariable			0.180		1	0.826			*0.031*
Low	153 (100)	1		56 (28)	0.85		97 (72)	1	
High	*15* (*12*)	*1.02* (*0.82–2.93*)		*5* (*2*)	(*0.20–3.59*)		*10* (*10*)	*2.09* (*1.07–4.09*)	
Multivariable			0.271			0.532			*0.022*
Low	153 (100)	1		56 (28)	1		97 (72)	1	
High	*15* (*12*)	*1.42* (*0.76–2.67*)		*5* (*2*)	*0.60* (*0.12–2.98*)		*10* (*10*)	*2.35* (*1.13–4.87*)	
CD68^+^ TAM									
Univariable			*0.029*			0.341			0.180
Low	137 (86)	1		55 (26)	1		82 (60)	1	
High	*29* (*24*)	*1.67* (*1.06–2.63*)		*6* (*4*)	*1.68* (*0.77–4.91*)		*23* (*20*)	*1.42* (*0.85–2.35*)	
Multivariable			0.102			0.501			0.125
Low	137 (86)	1		55 (26)	1		82 (60)	1	
High	29 (24)	1.48 (0.93–2.36)		6 (4)	1.63 (0.39–6.74)		23 (20)	1.52 (0.89–2.67)	
CD163^+^ TAM									
Univariable			*0.022*			0.174			0.346
Low	36 (17)	1		20 (7)	1		16 (10)	1	
High	*126* (*89*)	*1.84* (*1.09–3.09*)		*40* (*22*)	*1.81* (*0.77–4.24*)		*86* (*67*)	*1.38* (*0.71–2.68*)	
Multivariable			0.164			0.067			0.608
Low	36 (17)	1		20 (7)	1		16 (10)	1	
High	126 (89)	1.46 (0.85–2.57)		40 (22)	2.52 (0.94–6.81)		86 (67)	1.27 (0.58–2.58)	
MARCO^+^									
Univariable			0.195			*0.042*			0.718
Low	110 (*68*)	1		38 (14)	1		72 (54)	1	
High	*57* (*43*)	*1.29* (*0.88–1.89*)		*22* (*15*)	*2.14* (*1.03–4.44*)		*35* (*28*)	*1.09* (*0.69-1.72*)	
Multivariable			*0.002*		1	0.220		1	0.124
Low	110 (68)	1		38 (14)			72 (54)		
High	*57* (*68*)	*1.95* (*1.28–2.98*)		*22* (*15*)	*1.97* (*0.67–5.80*)		*35* (*28*)	*1.52* (*0.89–2.59*)	

Italics HRs and p values indicate significant values. Adjusted analysis included age (continuous), T-stage (1–2 vs 3–4), N-stage, differentiation grade (poor vs well- moderate), lymphatic invasion, vascular invasion, perineural growth, tumour morphology (I-type/PB-type) and adjuvant therapy (yes/no). Five cases were excluded due to neoadjuvant treatment, on the basis of death due to complications after surgical treatment or emigration

### Prognostic significance of CD68^+^ TAMs

**Fig. 3:**
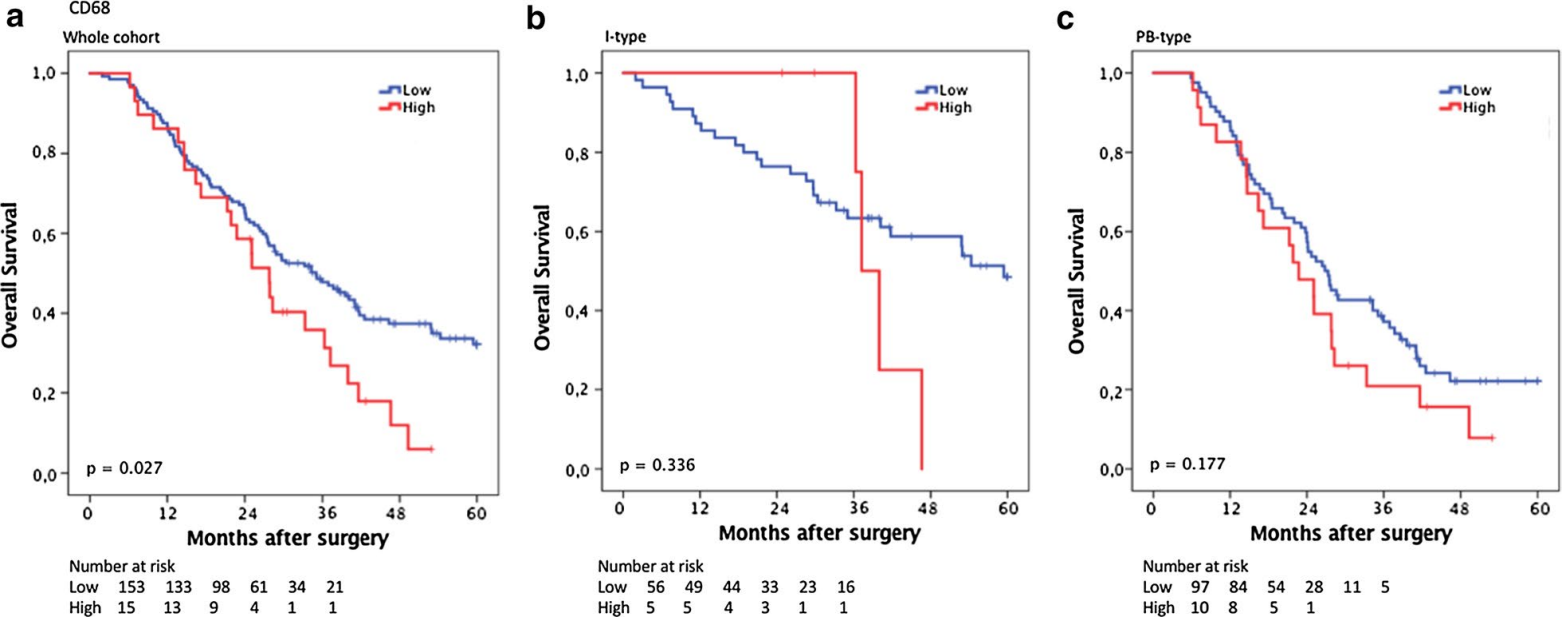
Kaplan–Meier estimates of survival according to CD68^+^ TAM density. Kaplan–Meier estimates of 5-year overall survival according to high and low CD68^+^ TAM density in **a** the entire cohort, **b** in I-type tumours and **c** in PB-type tumours

CRT analysis established an optimal prognostic cut off for total CD68^+^ cell infiltration at 126.75, whereby 141 cases were classified as having low CD68^+^ cell infiltration and 30 were classified as having high CD68^+^ infiltration. As shown in Fig. 3, Kaplan–Meier analysis of the whole cohort demonstrated a significantly shorter OS for patients with high infiltration of CD68^+^ cells (p = 0.027). This association did not remain significant when analysed in strata according to histological subtype, the trend was however similar in PB-type tumours (Fig. 3). As shown in Table 2, the association of high infiltration of CD68^+^ cells with a significantly reduced OS in the whole cohort was confirmed in unadjusted Cox regression analysis (HR = 1.67; 95% CI 1.06–2.63; p = 0.029), but did not remain significant in adjusted analysis. No significant associations with survival were seen when CD68^+^ macrophage density in stroma or tumour-nest was analysed separately, neither in the whole cohort nor in strata according to morphological type (data not shown). The prognostic significance of CD68^+^ macrophages did not differ significantly according to adjuvant chemotherapy (data not shown).

### Prognostic significance of CD163^+^ TAMs

**Fig. 4:**
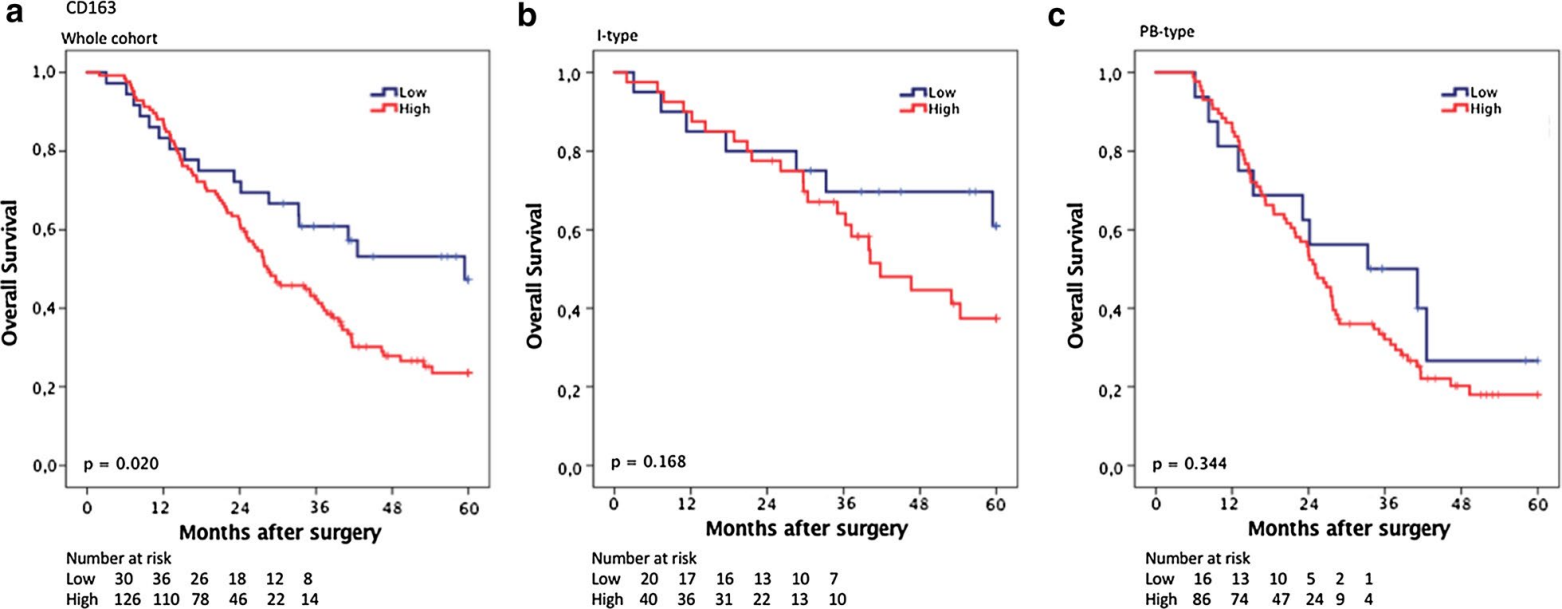
Kaplan–Meier estimates of survival according to CD163^+^ TAM density. Kaplan–Meier estimates of 5-year overall survival according to high and low CD163^+^ TAM density in **a** the entire cohort, **b** in I-type tumours and **c** in PB-type tumours

CRT analysis established an optimal prognostic cut off for total CD163^+^ cell infiltration at 104.5, whereby 37 cases were classified as having low CD163^+^ cell infiltration and 130 were classified as having high CD163^+^ cell infiltration. As shown in Fig. 4, Kaplan–Meier analysis of the whole cohort demonstrated a significantly shorter OS for patients with high levels of CD163^+^ cell infiltration (p = 0.020). This association did not remain significant when analysed in strata according to histological subtype, however the trend remained similar. As shown in Table 2, the association of high infiltration of CD163^+^ cells with a significantly reduced OS in the whole cohort was confirmed in unadjusted Cox regression analysis (HR = 1.84; 95% CI 1.09–3.09; p = 0.022) but did not remain significant in adjusted analysis. No significant associations with survival were seen when CD163^+^ macrophage density in stroma or tumour-nest was analysed separately, neither in the whole cohort nor in strata according to morphological type (data not shown). The prognostic significance of CD163^+^ macrophages did not differ significantly according to adjuvant chemotherapy (data not shown).

### Prognostic significance of MARCO^+^ TAM infiltration

**Fig. 5:**
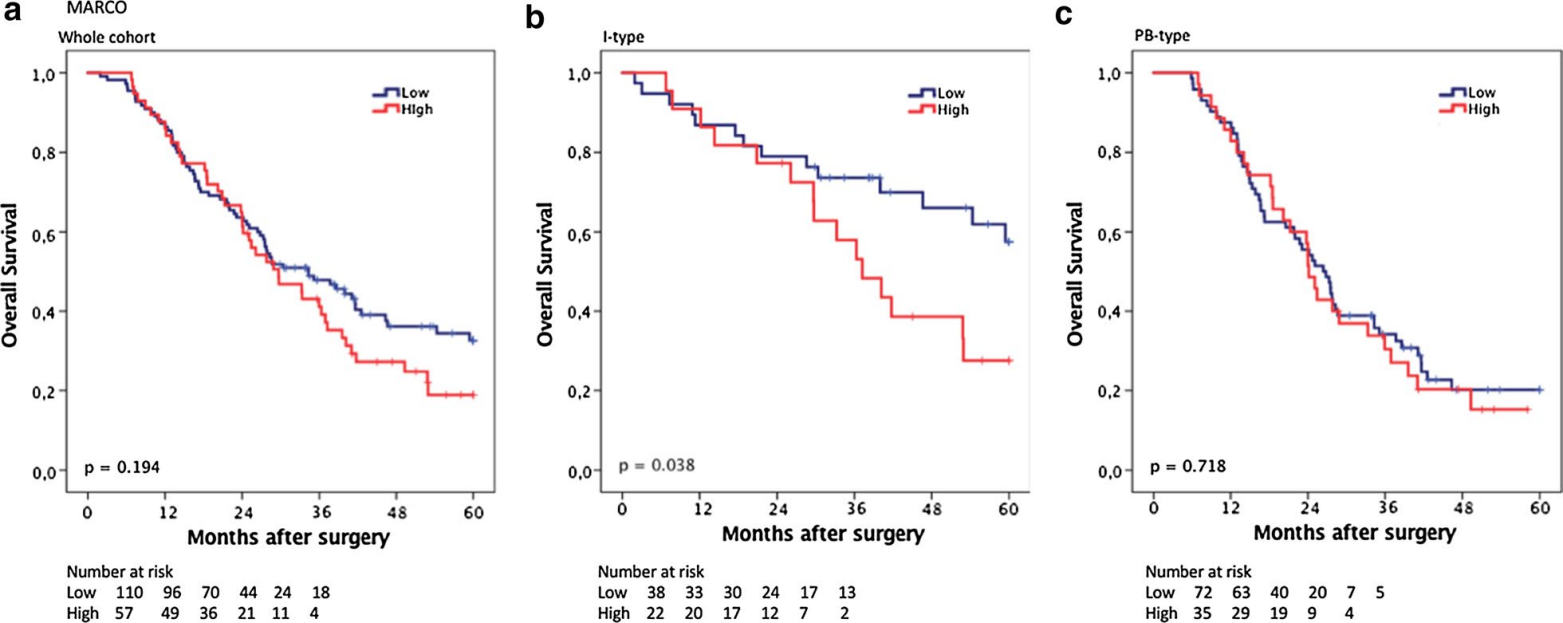
Kaplan–Meier estimates of survival according to MARCO^+^ TAM density. Kaplan–Meier estimates of 5-year overall survival according to high and low MARCO^+^ TAM density in **a** the entire cohort, **b** in I-type tumours and **c** in PB-type tumours

**Fig. 6:**
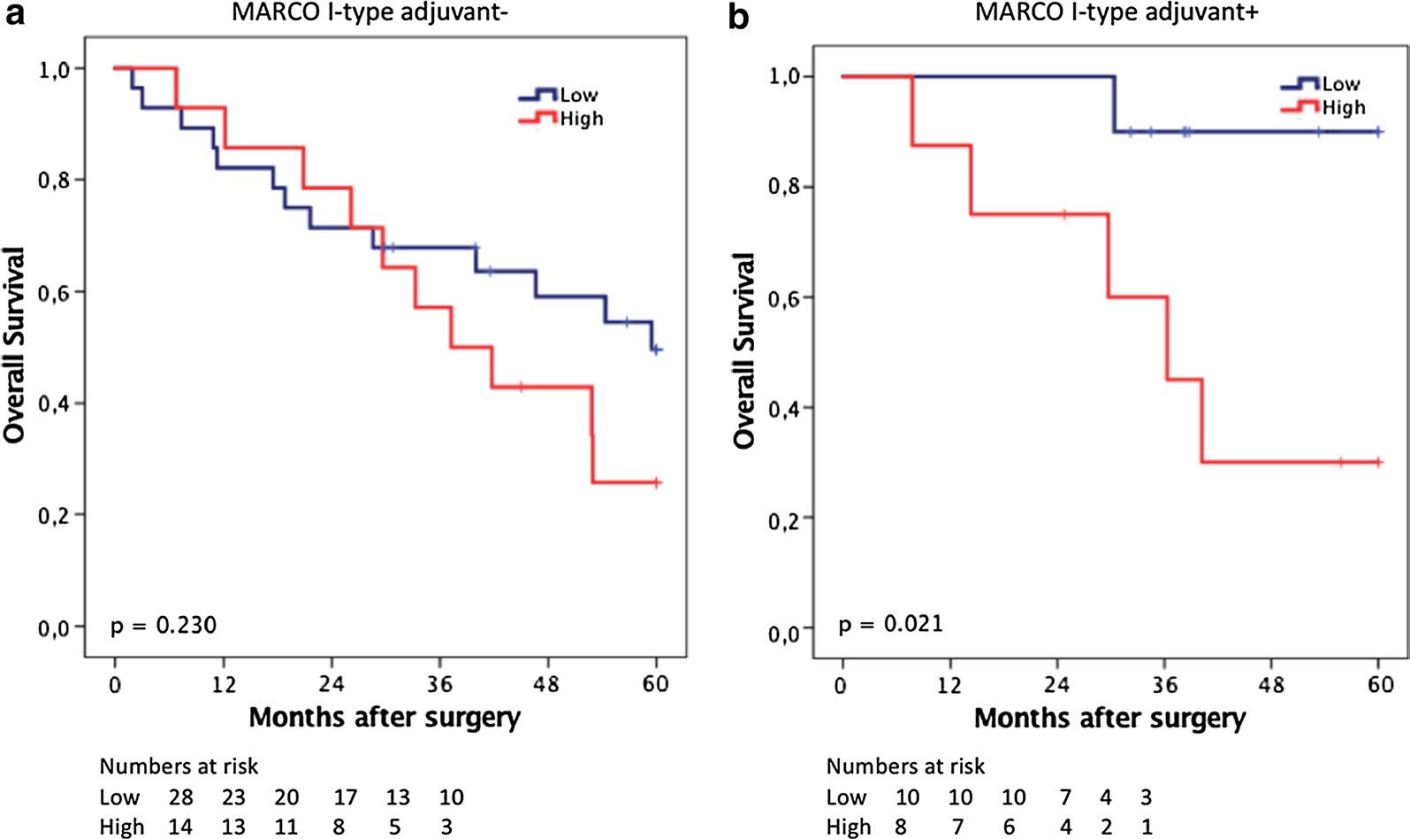
Kaplan–Meier estimates of survival according to MARCO^+^ TAM density and adjuvant chemotherapy. Kaplan–Meier estimates of 5-year overall survival according to high and low MARCO^+^ TAM density in patients with I-type tumours **a** not treated with adjuvant chemotherapy and **b** treated with adjuvant chemotherapy

CRT analysis established an optimal prognostic cut off for total MARCO^+^ cell infiltration at 8.5 whereby 115 cases were classified as having low MARCO^+^ cell infiltration and 57 were classified as having high MARCO infiltration. As shown in Fig. 5, MARCO was not associated with prognosis in the whole cohort (p = 0.194), or in PB-type tumours (p = 0.718). In I-type tumours, however, high MARCO^+^ density was significantly associated with a reduced OS (p = 0.038). This association was confirmed in unadjusted Cox regression analysis (HR = 2.14; 95% CI 1.03–4.44; p = 0.042), but did not remain significant in adjusted analysis (Table 2). Of note, in the entire cohort, high MARCO^+^ was significantly associated with a reduced OS in adjusted analysis (HR = 1.95; 95% CI 1.28–2.98; p = 0.002, Table 2). As shown in Fig. 6, in I-type tumours, high MARCO^+^ density was significantly associated with a reduced OS in patients who received adjuvant chemotherapy (p = 0.021), but not in patients who did not receive adjuvant chemotherapy. There was however no significant treatment interaction between MARCO^+^ and adjuvant chemotherapy (HR = 1.65 95% CI 0.72–3.79 for untreated vs HR = 8.46 95% CI 0.98–73.10, p_interaction_ = 0.165) in I-type tumours.

## Discussion

This study is, to our best knowledge, the first to investigate the prognostic role of tumour-infiltrating CD1a^+^, CD68^+^ and CD163^+^ and MARCO^+^ immune cells in periampullary adenocarcinoma, with particular reference to morphological type. The results demonstrate that high infiltration of CD1a^+^ TIDC is an independent predictor of a shorter survival in patients with PB-type tumours, but does not confer any prognostic value in patients with I-type tumours. These findings add to the growing evidence of a dysfunctional inflammatory microenvironment caused by a desmoplastic stroma in these particularly aggressive tumours.

A previous study failed to establish any association between survival and DC infiltration rates in pancreatic cancer, secondary to the scarcity of local homing [17]. The association between high CD1a^+^ TIDC density and poor prognosis may be explained by their immature state or even the induction of maturation defects in TIDC in situ, initiated by the tumour microenvironment and leading to an immunosuppressive phenotype encouraging tumour tolerance and immune evasion [24]. A study by Yamamoto et al. demonstrated a positive impact of TIDCs on survival in patients with pancreatic cancer [16]. However, in that study, anti-fascin was used as a marker for TIDCs, therefore potentially examining another TIDC population.

In the present study we chose to not investigate mature DCs or to identify functional subtypes of TIDCs. Future studies should however focus not only on the presence of tolerogenic immature CD1a^+^ DCs in tumour tissue, but also on their functional phenotype, as this may add important information on their potential immune modulatory effect in the inflammatory tumour microenvironment. There has been a steep increase of interest in the field of immunotherapy in recent years and dendritic cell vaccine has shown some promise in combination with conventional chemotherapy in pancreatic cancer [25–27]. The findings of the present study indicate that it is important to take morphological type into consideration when evaluating the results from such trials.

Moreover, this study demonstrated an association between high density of CD68^+^ and CD163^+^ TAMs with poor prognosis in the whole cohort, but not in strata according to morphological type, and not independent of established clinical prognostic factors. Tumour-educated TAMs facilitate progression of pancreatic cancer and promote angiogenesis, remodelling of stroma, epithelial-mesenchymal transition and extravasation of tumour cells [28, 29]. Previous studies have demonstrated that TAMs are associated with poor prognosis in pancreatic cancer [7, 8], which is in line with the findings of the present study encompassing the full spectrum of periampullary adenocarcinoma. Even though it is mainly the anti-inflammatory subpopulation of TAMs that has been suggested to promote tumour progression, the entire CD68^+^ TAM population was also found to be associated with poor prognosis in the present study. The reason for this finding is most likely that the predominant subtype of TAM in the tumour microenvironment is leaning towards pro-tumour polarisation [30], thus making up for a large part of the CD68^+^ TAM population.

Further, the novel macrophage marker MARCO, which has been shown to be a target for immunotherapy [14], was found to be associated with poor prognosis in I-type but not PB-type tumours. The observation that the prognostic impact of MARCO^+^ cells was particularly evident in patients who received adjuvant chemotherapy, as opposed to patients who did not receive any adjuvant treatment, is noteworthy, despite the lack of a significant treatment interaction. The OS of patient with low MARCO^+^ TAM infiltration who received adjuvant chemotherapy with I-type morphology had a remarkably better OS than patients that did not receive adjuvant chemotherapy. This result might indicate a potential predictive role of MARCO^+^ TAM infiltration for chemotherapy response, or high density of MARCO^+^ TAMs could be a sign of chemotherapy resistance. Further studies are needed to validate these results, especially in intestinal cancers.

Previous research on the role of MARCO in cancer has been scarce. One previous study on hepatocellular cancer showed that decreased expression of MARCO was associated with poor prognosis [31], however, in contrast to the present study, that study did not look at immune cell specific expression of MARCO, but rather at intra-tumoural MARCO expression. The present study found a significant association between MARCO^+^ cells and CD68^+^ cells which confirms the results of Sun et al, where MARCO^+^ cells co-localized with CD68^+^ macrophages [31]. If the association between high MARCO^+^ immune cell infiltration and poor survival rates is due to the co-localization of MARCO^+^ and CD68^+^ cells, interaction with chemotherapy, or because of the biological mechanisms of MARCO is yet to be determined. Further research into the role of MARCO in periampullary/pancreatic cancer as well as in other intestinal cancer and a broader spectrum of solid cancers is highly warranted.

In a translational context, the findings from the analyses of human tumours in this study are well in line with previous in vitro studies. For instance, Karnevi et al. have shown an intricate interplay between macrophages and tumour cells in vitro, where tumour derived factors drive the differentiation of macrophages into a pro-tumour phenotype [30]. Additionally, the opposite effect has been demonstrated for dendritic cells in vitro, where tumour cell derived factors inhibit and limit the normal anti-tumour function of DCs [32]. Further, in vivo models have shown that macrophage infiltration increases with tumour progression, and that infiltration starts very early before any invasive potential has been developed by the tumour. Collectively, these findings support the conclusions in the present study, wherein dense infiltration of TAMs and DCs are shown to be associated with poor and improved prognosis, respectively, in periampullary adenocarcinoma.

Some subgroup analyses rendered rather small numbers of cases, in particular in strata according to adjuvant or no adjuvant therapy and morphological type. Therefore, the results from the present study need to be validated in additional and preferably larger patient cohorts. However, as about half of the patients in the herein analysed patient cohort received adjuvant chemotherapy and half of the patients did not, it may give some indications to the potential predictive value of the investigated biomarkers, despite the retrospective setting.

Another potential limitation to the study is the use of the TMA technique, and in particular the fact that the tissue cores were primarily sampled from areas with tumour and not the adjacent stroma. However, a large proportion of periampullary cancers have a comparatively high stromal/tumour cell ratio, and three 1 mm cores can be considered a generous sampling. However, validating studies should ideally be specifically designed for a more comprehensive mapping of immune cell signatures. In this context, the TMA technique is likely to be superior to whole tissue section analysis, since it allows for sampling of multiple tissue types from multiple tissue blocks, and thus for a more comprehensive analysis of the inflammatory microenvironment of individual tumours.

## Conclusions

This study provides a first description of an independent prognostic value of tolerogenic, immature dendritic cells in periampullary adenocarcinoma, especially in PB-type tumours. Further, while high CD68^+^ and CD163^+^ TAM density was associated with poor survival rates in the whole cohort, although not independent of other factors, high MARCO^+^ TAM density was associated with lower survival rates in I-type tumours, and an independent factor of poor prognosis in the entire cohort. Moreover, the prognostic value of MARCO^+^ TAMs was only evident in patients treated with adjuvant chemotherapy. These findings provide further insight into the complexity of the role of the immune system in the tumour microenvironment of periampullary adenocarcinoma, including pancreatic cancer, and further emphasize the importance of taking tumour morphology rather than anatomic location into consideration in biomarker studies and when evaluating results from clinical trials.

## Additional files

**Additional file 1.** Associations between CD1a^+^ DC-cell infiltration and clinicopathological factors.

**Additional file 2.** Associations between CD68^+^ infiltration and clinicopathological factors.

**Additional file 3.** Associations between CD163^+^ TAM infiltration and clinicopathological factors.

**Additional file 4.** Associations between MARCO^+^ infiltration and clinicopathological factors.

## Abbreviations

APC: antigen presenting cell
CI: confidence interval
CRT: classification and regression tree
DC: dendritic cell
HR: hazard ratio
I-type: intestinal type
IHC: immunohistochemistry
MARCO: macrophage receptor with collagenous structure
NK-cell: natural killer cell
NKT-cell: natural killer T cell
PAMP: pathogen associated molecular patterns
PB-type: pancreatobililary type
OS: 5-year overall survival
TAM: tumour-associated macrophages
TIDC: tumour-infiltrating dendritic cells
TLR: toll-like receptor
TMA: tissue microarray
