# Supplementary material for: The clinical importance of tumour-infiltrating macrophages and dendritic cells in periampullary adenocarcinoma differs by morphological subtype
Source: J Transl Med. 2017 Jul 3;15:152. doi: 10.1186/s12967-017-1256-y (PMC5496326; doi:10.1186/s12967-017-1256-y)
Supplement: Supplementary file 1 — Additional file 1. Associations between CD1a+ DC-cell infiltration and clinicopathological factors. [file 12967_2017_1256_MOESM1_ESM.docx]

Additional file 1: Associations between CD1a^+^ DC-cell infiltration and clinicopathological factors.

|  | Pancreatobiliary-type | | Intestinal-type | |
| --- | --- | --- | --- | --- |
| Factor (n = PB-type; n = I-type) | Total CD1a median (range) | p-value | Total CD1a median (range) | p-value |
| Age* Q1 (n = 20; n = 18) Q2 (n = 31; n = 13) Q3 (n = 25; n = 18) Q4 (n = 31; n = 12) | 0.00 (0.00-3.50)  1.00 (0.00-4.00) 0.50 (0.00-4.00) 0.00 (0.00-3.00) | **0.011** | 0.00 (0.00-3.00) 0.00 (0.00-6.00) 0.00 (0.00-4.0) 0.00 (0.00-2.50) | 0.817 |
| Sex Female (n = 51; n = 34) Male (n = 57; n = 29) | 0.00 (0.00-4.00) 0.00 (0.00-4.00) | 0.771 | 0.00 (0.00-4.00) 0.00 (0.00-6.00) | 0.052 |
| Differentiation grade Well (n = 7; n = 5) Moderate (n = 33; n = 26) Poor (n = 64; n = 32) Undifferentiated (n =4; n = 0) | 0.00 (0.00-2.00)  0.00 (0.00-4.00) 0.25 (0.00-4.00) 0.25 (0.00-1.00) | 0.898 | 0.00 (0.00-1.00) 0.00 (0.00-4.00) 0.00 (0.00-6.00) | 0.438 |
| Tumour stage T1 and T2 (n = 13; n = 15)  T3 and T4 (n = 95; n = 48) | 0.00 (0.00-4.00) 0.00 (0.00-4.00) | 0.854 | 0.00(0.00-4.00) 0.00 (0.00-6.00) | 0.512 |
| Nodal stage N0 (n = 31; n = 33) N1 (n =45; n = 19) N2 (n = 32, n = 11) | 0.00 (0.00-4.00) 0.00 (0.00-4.00) 1.00 (0.00-4.00) | 0.186 | 0.00 (0.00-6.00) 0.00 (0.00-4.00) 0.00 (0.00-2.00) | 0.362 |
| Resection margins R0 (n = 7; n = 17) R1 (n = 79; n = 14) RX (n = 22; n = 32) | 1.00 (0.00-2.00) 0.00 (0.00-4.00) 0.00 (0.00-4.00) | 0.537 | 0.00 (0.00-4.00) 0.00 (0.00-2.00) 0.00 (0.00-6.00) | 0.758 |
| Perineural growth Absent (n = 23; n = 44) Present (n = 85, n = 19) | 0.00 (0.00-2.00) 1.00 (0.00-4.00) | 0.094 | 0.00 (0.00-4.00)  0.00 (0.00-6.00) | 0.116 |
| Lymphatic growth Absent (n = 33; n = 29) Present (n = 75; n = 34) | 1.00 (0.00-4.00) 0.00 (0.00-4.00) | 0.817 | 0.00 (0.00-4.00) 0.00 (0.00-6.00) | 0.407 |
| Vascular growth Absent (n = 71; n = 58) Present (n = 37; n = 5) | 0.50 (0.00-4.00) 0.00 (0.00-4.00) | 0.287 | 0.00 (0.00-6.00) 0.00 (0.00-1.00) | 0.480 |
| Peripancreatic fat growth Absent (n = 23; n = 41) Present (n = 85; n = 22) | 0.00 (0.00-4.00) 0.50 (0.00-4.00) | 0.401 | 0.00 (0.00-4.00) 0.00 (0.00-6.00) | 0.535 |

* Q1 = 38-61, Q2 = 62-67, Q3 = 68-72, Q4 = 73-84
