# Supplementary material for: The clinical importance of tumour-infiltrating macrophages and dendritic cells in periampullary adenocarcinoma differs by morphological subtype
Source: J Transl Med. 2017 Jul 3;15:152. doi: 10.1186/s12967-017-1256-y (PMC5496326; doi:10.1186/s12967-017-1256-y)
Supplement: Supplementary file 2 — Additional file 2. Associations between CD68+ infiltration and clinicopathological factors. [file 12967_2017_1256_MOESM2_ESM.docx]

Additional file 2: Associations between CD68^+^ infiltration and clinicopathological factors.

|  | Pancreatobiliary-type | | Intestinal-type | |
| --- | --- | --- | --- | --- |
| Factor (n =PB-type; n = I-type) | Total CD68 median (range) | p-value | Total CD68 median (range) | p-value |
| Age* Q1 (n = 19; n = 18) Q2 (n = 31; n = 10) Q3 (n = 25; n = 17) Q4 (n = 30; n = 11) | 25.00 (19.00-229.50)  100.00 (27.00-230.00) 96.00 (36.00-186.00) 90.50 (25.00-210.00) | 0.418 | 92.00 (19.00-350.00) 68.50 (20.00-142.00) 71.00 (28.00-85.50) 99.00 (29.00-220.50) | 0.237 |
| Sex Female (n = 49; n = 31) Male (n = 57; n = 27) | 90.00 (27.00-210.00) 112.50 (25.00-230.00) | 0.141 | 74.00 (19.00-350.00) 96.50 (30.50-220.50) | **0.044** |
| Differentiation grade Well (n = 7; n = 5) Moderate (n = 33; n = 25) Poor (n = 62; n = 28) Undifferentiated (n =4; n = 0) | 88.00 (46.00-108.00)  103.00 (25.00-229.50) 90.50 (27.00-230.00) 138.00 (100.00-171.00) | 0.228 | 125.00 (71.00-148.00) 96.50 (28.00-220.50) 72.00 (19.00-350.00) | 0.438 |
| Tumour stage T1 and T2 (n = 12; n = 14)  T3 and T4 (n = 94; n = 44) | 84.00 (25.00-199.00) 99.00 (27.00-230.00) | 0.414 | 86.00(46.00-185.50) 86.50 (19.00-350.00) | 0.398 |
| Nodal stage N0 (n = 30; n = 30) N1 (n =45, n = 18) N2 (n = 31, n = 10) | 92.50 (36.00-229.50) 91.00 (25.00-230.00) 114.00 (41.00-210.00) | 0.097 | 77.00 (19.00-185.50) 78.25 (35.00-200.00) 96.50 (46.00-220.50) | 0.152 |
| Resection margins R0 (n = 7; n = 16) R1 (n = 77;, n = 14) RX (n = 22; n = 28) | 101.50 (53.50-133.00) 100.00 (25.00-230.00) 91.25 (51.00-191.00) | 0.978 | 87.00 (19.00-180.00) 91.75 (35.00-350.00) 75.00 (20.00-185.50) | 0.557 |
| Perineural growth Absent (n = 23; n = 44) Present (n = 83, n = 18) | 92.00 (36.00-197.00) 100.50 (25.00-230.00) | 0.321 | 78.00 (19.00-185.50)  93.25 (35.00-350.00) | 0.278 |
| Lymphatic growth Absent (n = 32; n = 28) Present (n = 74; n = 30) | 91.50 (27.00-230.00) 100.25 (25.00-210.00) | 0.399 | 91.75 (20.00-200.50) 81.50 (19.00-350.00) | 0.494 |
| Vascular growth Absent (n = 71; n = 53) Present (n = 35; n = 5) | 104.50 (25.00-230.00) 79.50 (27.00-210.00) | **0.018** | 87.00 (19.00-350.00) 41.00 (30.50-100.00) | 0.138 |
| Peripancreatic fat growth Absent (n = 22; n = 38) Present (n = 84; n = 20) | 91.50 (30.50-229.50) 100.75 (25.00-230.00) | 0.398 | 73.50 (19.00-185.50) 93.25 (35.00-350.00) | 0.087 |

* Q1 = 38-61, Q2 = 62-67, Q3 = 68-72, Q4 = 73-84
