# Supplementary material for: The clinical importance of tumour-infiltrating macrophages and dendritic cells in periampullary adenocarcinoma differs by morphological subtype
Source: J Transl Med. 2017 Jul 3;15:152. doi: 10.1186/s12967-017-1256-y (PMC5496326; doi:10.1186/s12967-017-1256-y)
Supplement: Supplementary file 3 — Additional file 3. Associations between CD163+ TAM infiltration and clinicopathological factors. [file 12967_2017_1256_MOESM3_ESM.docx]

Additional file 3: Associations between CD163^+^ TAM infiltration and clinicopathological factors.

|  | Pancreatobiliary-type | | Intestinal-type | |
| --- | --- | --- | --- | --- |
| Factor (n = PB-type; n = I-type) | Total CD163 median (range) | p-value | Total CD163 median (range) | p-value |
| Age* Q1 (n = 19; n = 18) Q2 (n = 31; n = 12) Q3 (n = 25; n = 18) Q4 (n = 29; n = 12) | 140.00 (70.00-200.00)  140.00 (68.00-250.00) 139.50 (85.00-275.00) 130.00 (47.00-220.00) | 0.985 | 125.00 (57.00-220.00) 122.50 (35.00-158.00) 120.00 (37.00-200.00) 155.00 (63.00-250.00) | 0.294 |
| Sex Female (n = 49; n = 33) Male (n = 54; n = 29) | 130.00 (47.00-250.00) 140.00 (49.00-275.00) | 0.622 | 120.00 (35.00-120.00) 130.00 (57.00-250.00) | 0.225 |
| Differentiation grade Well (n = 7; n = 5) Moderate (n = 32; n = 26) Poor (n = 60; n = 31) Undifferentiated (n =4; n = 0) | 127.00 (47.00-185.00)  140.25 (59.00-250.00) 136.50 (49.00-200.00) 185.00 (145.00-275.00) | 0.139 | 150.00 (100.00-250.00) 130.00 (35.00-220.00) 120.00 (37.00-200.00) | 0.438 |
| Tumour stage T1 and T2 (n = 12; n = 14)  T3 and T4 (n = 91; n = 48) | 131.25 (47.00-250.00) 139.25 (49.00-275.00) | 0.575 | 140.50(63.00-250.00) 123.00 (35.00-220.00) | 0.474 |
| Nodal stage N0 (n = 30; n = 32) N1 (n =43; n = 19) N2 (n = 30; n = 11) | 141.00 (47.00-250.00) 130.00 (59.00-275.00) 140.00 (68.00-200.00) | 0.689 | 130.00 (35.00-250.00) 128.00 (48.00-200.00) 130.00 (57.00-190.00) | 0.940 |
| Resection margins R0 (n = 7; n = 17) R1 (n = 75;, n = 14) RX (n = 21; n = 31) | 140.00 (47.00-170.00) 135.00 (59.00-275.00) 160.00 (49.00-250.00) | 0.325 | 137.00 (37.00-220.00) 112 (48.00-200.00) 125.00 (35.00-250.00) | 0.547 |
| Perineural growth Absent (n = 23; n = 44) Present (n = 80; n = 19) | 138.00 (47.00-275.00) 139.75 (49.00-220.00) | 0.968 | 130.00 (35.00-200.00)  120.00 (48.00-200.00) | 0.396 |
| Lymphatic growth Absent (n = 30; n = 29) Present (n = 73; n = 34) | 134.75 (47.00-275.00) 140.00 (49.00-220.00) | 0.687 | 120.00 (35.00-220.00) 130.00 (48.00-250.00) | 0.719 |
| Vascular growth Absent (n = 71; n = 57) Present (n = 34; n = 5) | 140.00 (47.00-250.00) 130.00 (49.00-275.00) | 0.053 | 130.00 (35.00-250.00) 93.00 (48.00-130.00) | 0.083 |
| Peripancreatic fat growth Absent (n = 22; n = 42) Present (n = 81; n = 22) | 131.50 (47.00-250.00) 140.00 (49.00-275.00) | 0.955 | 127.50 (35.00-250.00) 125.50 (48.00-200.00) | 0.895 |

* Q1 = 38-61, Q2 = 62-67, Q3 = 68-72, Q4 = 73-84
