# Supplementary material for: The clinical importance of tumour-infiltrating macrophages and dendritic cells in periampullary adenocarcinoma differs by morphological subtype
Source: J Transl Med. 2017 Jul 3;15:152. doi: 10.1186/s12967-017-1256-y (PMC5496326; doi:10.1186/s12967-017-1256-y)
Supplement: Supplementary file 4 — Additional file 4. Associations between MARCO+ infiltration and clinicopathological factors. [file 12967_2017_1256_MOESM4_ESM.docx]

Additional file 4: Associations between MARCO^+^ infiltration and clinicopathological factors.

|  | Pancreatobiliary-type | | Intestinal-type | |
| --- | --- | --- | --- | --- |
| Factor (n = PB-type; n = I-type) | Total MARCO median (range) | p-value | Total MARCO median (range) | p-value |
| Age* Q1 (n = 20; n = 18) Q2 (n = 31; n = 13) Q3 (n = 25; n = 18) Q4 (n = 31; n = 11) | 3.50 (00.00-35.00)  7.00 (00.00-30.00) 7.00 (00.00-43.00) 4.00. (00.00-69.00) | 0.231 | 3.50 (00.00-65.00) 4.00 (00.00-78.00) 5.00 (00.00-25.00) 7.00 (00.00-48.00) | 0.589 |
| Sex Female (n = 51; n = 34) Male (n = 57; n = 28) | 5.00 (00.00-69.00) 4.00 (00.00-43.00) | 0.673 | 6.00 (00.00-65.00) 4.00 (4.00-78.00) | 0.943 |
| Differentiation grade Well (n = 7; n = 5) Moderate (n = 33; n = 25) Poor (n = 64; n = 32) Undifferentiated (n =4; n = 0) | 4 (00.50-8.00)  5.00 (00.00-43.00) 5.00 (00.00-64.00) 14.75 (1.00-28.00) | 0.380 | 7.00 (1.00-11.00) 4.00 (00.00-48.00) 4.50 (00.00-78.00) | 0.771 |
| Tumour stage T1 and T2 (n = 13; n = 15)  T3 and T4 (n = 95; n = 47) | 5.00 (1.00-27.00) 5.00 (0.00-69.00) | 0.660 | 4.00 (00.00-25.00) 5.00 (00.00-78.00) | 0.562 |
| Nodal stage N0 (n = 31; n = 32) N1 (n =45; n = 19) N2 (n = 32; n = 11) | 5.00 (0.00-69.00) 5.00 (0.00-28.00) 5.50 (00.00-43.00) | 0.910 | 6.75 (00.00-78.00) 3.00 (00.00-65.00) 4.00 (0.00-67.00) | 0.178 |
| Resection margins R0 (n = 7; n = 16) R1 (n = 79; n = 14) RX (n = 22; n = 32) | 4.00 (1.00-14.00) 5.00 (00.00-39.00) 5.50 (0.00-69.00) | 0.789 | 5.50 (0.00-26.00) 2.50 (00.00-65.00) 6.00 (0.00-78.00) | 0.579 |
| Perineural growth Absent (n = 23; n = 43) Present (n = 85; n = 19) | 10 (47.00-275.00) 5.00 (0.00-43.00) | 0.769 | 5.00 (0.00-67.00)  4.00 (0.00-78.00) | 0.438 |
| Lymphatic growth Absent (n = 33; n = 28) Present (n = 75; n = 34) | 5.00 (0.00-35.00) 4.50 (0.00-69.00) | 0.512 | 4.25 (00.00-48.00) 5.50 (0.00-78.00) | 0.588 |
| Vascular growth Absent (n = 71; n = 57) Present (n = 37; n = 5) | 7.00 (0.00-69.00) 2.00 (0.00-43.00) | **<0.001** | 5.00 (0.00-78.00) 2.00 (0.00-16.00) | 0.324 |
| Peripancreatic fat growth Absent (n = 23; n = 41) Present (n = 85; n = 21) | 4.50 (0.00-69.00) 5.00 (0.00-43.00) | 0.934 | 4.50 (0.00-34.00) 5.00 (0.00-78.00) | 0.288 |

* Q1 = 38-61, Q2 = 62-67, Q3 = 68-72, Q4 = 73-84
